# Synergistic influence of probiotic and florfenicol on embryonic viability, performance, and multidrug-resistant *Salmonella* Enteritidis in broiler chickens

**DOI:** 10.1038/s41598-023-36238-6

**Published:** 2023-06-14

**Authors:** Nehal M. Nabil, Maram M. Tawakol, Abdelhafez Samir, Heba M. Hassan, Ahlam E. Yonis, Reem M. Reda, Mona M. Elsayed

**Affiliations:** 1 Reference Laboratory for Veterinary Quality Control On Poultry Production, Animal Health Research Institute, Agricultural Research Center (ARC), Nadi El-Seid Street, Dokki, 12618 Giza Egypt; 2 Department of Hygiene and Zoonoses, Faculty of Veterinary Medicine, Mansoura University, Mansoura, 35516 Egypt

**Keywords:** Microbiology, Zoology

## Abstract

This study was conducted during the period of August 2021 to April 2022 and divided into two parts. The first part involved the isolation and characterization of *Salmonella* from 200 diseased broiler chickens collected from farms in Dakahlia Governorate, Egypt, with the detection of its antimicrobial susceptibility. The second experimental part involved in ovo inoculation of probiotics and florfenicol to evaluate their effects on hatchability, embryonic viability, growth performance traits and the control of multidrug-resistant *Salmonella* Enteritidis infections post hatching. The point prevalence of *Salmonella* in the internal organs of diseased chickens was 13% (26/200), including 6 serotypes: *S.* Enteritidis*, S.* Typhimurium*, S.* Santiago*, S.* Colindale*, S.* Takoradi* and S.* Daula. Multidrug resistance was found in 92% (24/26) of the isolated strains with a multiantibiotic resistance index of 0.33–0.88 and 24 antibiotic resistance patterns. The in ovo inoculation of probiotic with florfenicol showed significant improvement in the growth performance parameters compared with other groups and had the ability to prevent colonization of multidrug resistant *S.* Enteritidis in the majority of the experimental chicks, and the remaining chicks showed very low colonization, as detected by RT‒PCR. These findings suggested the application of in ovo inoculation techniques with both probiotics and florfenicol as a promising tool to control multidrug-resistant *S.* Enteritidis in poultry farms.

## Introduction

*Salmonella* enterica is considered one of the most important foodborne pathogens^1,2^, producing a global economic impact on public health^3^. Annually, there are approximately 93 million cases of human gastroenteritis and up to 155,000 deaths due to *Salmonella* infections worldwide^1^. Poultry and poultry products are the main important sources of human *Salmonella* infections^1,4^, and most outbreaks are caused by chickens, as they are known to be carriers of *Salmonella* in their gut. According to European Union One Health Zoonoses Report during 2019, broiler was the source of 70.0% of all serotyped *Salmonella* isolates^5^. *S.* Enteritidis infecting poultry-producing fowl paratyphoid^6^ and associated with the occurrence of human salmonellosis during the last 20 years^7^. *Salmonella* is still a major problem facing the poultry industry, not only because of the risk arising from mortalities but also because of the economic losses and reduced efficiency that can occur as a result of bird infection^8^. Unfortunately, pathogens can infect chicks while they are still at the hatchery, throughout the process of hatching, sexing, receiving vaccinations, and being transported and even before they have had their first feed^9^. In current poultry production systems, the lack of contact between chicks and adult birds slows the colonization of the intestinal tract by beneficial microbes^10^. Initial microbial colonization is crucial to promote immune system growth and maturation as well as to prevent harmful bacteria from colonizing by competitive exclusion^11^. Particularly in newborn animals with rapid growth rates, gut health and function are crucial for supplying all essential nutrients for growth and maintenance^12^.

The elimination of drugs used as growth promoters in animal diets^13^ or in the treatment of different pathogens from the most important points must be considered to avoid the misuse of antibiotics, which facilitates the generation of antibiotic resistance genes in bacteria infecting poultry farms^14^. The emergence of multidrug resistance in *Salmonella* isolates affecting the food chain has become a public health concern worldwide^15^. Therefore, many approaches should be implemented to control *Salmonella* and other intestinal bacteria infecting poultry, such as in ovo inoculation with probiotics^16^, which not only improves the intestinal epithelium, immune system and health condition of birds^17^ but also increases egg hatchability, reduces embryonic mortality and increases posthatch growth performance^18^. Probiotics are live cultures of microbes that have beneficial effects on host health. Its application in poultry diets improves the birds’ immune responses, performance, and feed conversion ratios^19^ and prevents enteric pathogens by the competitive exclusion mechanism^17^. The use of probiotics as feed additives improves the quality and taste of poultry products^20^. On the other hand, amphenicol drugs have an increased role as therapeutic agents against avian pathogens. They have bacteriostatic effects and a wide antibacterial spectrum. Most gram-positive and gram-negative organisms are susceptible^21^. Florfenicol belongs to the amphenicol pharmacological group and is approved in the European Union for use in cattle, sheep, pigs and chickens^22^.

There is little information available about the effects of injecting florfenicol injectable solution into chicken eggs. The injection of florfenicol in embryonated chicken eggs showed no toxicity and no gross abnormality in the tissues and external body of the embryos, and it can be used for the success of the eradication scheme^23^. Therefore, this study aims to isolate and characterize *S.* Enteritidis from diseased birds and for the first time investigate the synergistic influence of in ovo-inoculation of probiotic and florfenicol on embryonic viability, growth performance, and the control of multidrug-resistant *S*. Enteritidis in broiler chicks.

## Materials and methods

### Sample collection

A total of 200 diseased broiler chickens (age: 22 to 32 days) were collected randomly from 10 farms located in Dakahlia Governorate, Egypt. The collected chickens suffered from watery diarrhea, inappetence, poor growth, ruffled feathers and weakness. The birds were subjected to post-mortem examinations (PM), and samples from internal organs (liver, spleen, heart and cecum) were collected aseptically and then labelled and transported directly to the Reference Laboratory for Veterinary Quality control on Poultry production for further examinations.

This protocol was performed by following the animal ethics guidelines and approved by the Medical Research Ethics Committee of Mansoura University with code number [R/143]. Additionally, all methods are reported in accordance with International Guiding Principles for Biomedical Research Involving Animals as issued by the Council for the International Organizations of Medical Sciences.

### *Salmonella* isolation and identification

*Salmonella* was isolated according to^24^. Briefly, the internal organs from each bird were pooled together and considered one sample. One gram of each sample was added to 9 ml of nutrient broth (Oxoid, UK) and incubated at 37 °C for 18 h. After pre-enrichment, 1 ml of each broth culture was transferred to 9 ml of Selenite F broth (Oxoid, UK) and incubated at 37 °C for 18 h. A loop-full of culture from Selenite F broth was streaked into plates of XLD (Oxoid, UK). The plates were incubated at 37 °C for 18 h and checked for growth of typical colonies of *Salmonella spp*. Conventional biochemical methods including the urase test, triple sugar iron (TSI), lysine decarboxylase test, ornithin decarboxylase test, indole test, citrate utilization test and xylose, sucrose, rhamnose and arabinose fermentation tests were performed according to^25^. According to the Kauffman-White scheme^26^, the serological identification of *Salmonella* isolates for the detection of somatic and flagellar antigens was performed.

### Antimicrobial sensitivity

*Salmonella* isolates were tested against 9 antibiotics commonly used in broiler farms in Egypt by the disc diffusion method^27^. Nine antibiotics belonging to 6 classes were selected. They included Sulfonamides such as sulfamethoxazole-trimethoprim (25 µg), Tetracyclines such as oxytetracycline (OT; 30 µg), doxycycline (DO; 30 µg), Aminoglycosides such as streptomycin (S; 10 µg), neomycin (N; 30 µg), penicillin ampicillin/sulbactam (SAM; 20 µg), and amoxicillin (AMX; 10 µg), Polypeptides such as colistin sulfate (CT; 25 µg) and Amphotericols such as florfenicol (FFC; 30 µg). The recorded inhibition zones were interpreted according to^28^.

### In vivo inoculation of probiotic in SPF embryonated chicken egg and its role in the control of *Salmonella* posthatching

*Experimental design*: Specific pathogen-free (SPF) embryonated chicken eggs (n = 75) were obtained from a Ross broiler breeder flock (age = 38 weeks) that was free from *Salmonella* and not vaccinated. The collected eggs were individually weighed and divided into 5 groups (15 eggs for each) with similar average egg weights for each group. The eggs were then incubated under standard conditions (54% relative humidity and 37.5 °C)^16^.

The embryo viability was checked throughout the incubation period; all eggs were candled to discard any unfertile eggs and dead embryos. After the egg candling process on the 18th day of incubation, the eggs were transported outside the incubator, disinfected with 70% alcohol and inoculated as follows: groups (1) and (2) were control groups inoculated with phosphate buffered saline, and group (3) was inoculated with a commercial probiotic bacterial product (Wiser biotic, WISER CARE company, Egypt, batch no: 100210117). Each egg in group (3) was inoculated with 0.3 ml of sterile saline solution containing 1 gm of the probiotic product containing Bacillus subtilis 5.25 × 10^11^ CFU and Bacillus licheniforms 5.25 × 1011 CFU for each kg)^18^ in the air sac using a pipette attached to a 23-ga needle, and group (4) was injected with florfenicol product (Floricol, PHARMA SWEDE company, Egypt Batch no: L201226) (each ml contained 100 mg florfenicol). A total of 0.5 ml saline solution containing 20 mg florfenicol for each kg egg weight)^29^. Group (5) was inoculated with both probiotic bacterial solution and florfenicol as mentioned above. After the inoculation process finished, all holes in the inoculated eggs were immediately sealed, and the eggs were returned again to the incubator. At day 21 (hatching day), the hatchability percentage was determined, and all non-hatched eggs were examined to explore the cause of embryo death.

The post hatch effects of probiotic, florfenicol and probiotic mixture with florfenicol in ovo inoculation of *S.* Enteritidis infection were carried out as follows: 50 hatched chicks were selected from the previous 5 egg groups (10 chicks from each group), and the groups kept their previous numbers. The experiment was conducted in the biosafety level 2 + animal facility (BSL2 +). Chicks housed in separate rooms in metal grid cages. Water and feed were provided ad libitum during the whole experiment period. The temperature was manually controlled and gradually reduced from 32 to 33 °C in the first week to 20–21 °C at the end of the experiment. Chickens were first maintained on a 24-h light cycle, and then the light program was progressively changed to 18 h of light and 6 h of darkness. The number of air exchanges was 12 per hour and humidity was maintained between 55 and 70%. Chickens not vaccinated. These chicks were challenged orally at the 2nd day of age with a multidrug-resistant *S.* Enteritidis strain (selected from the isolates of this study) at 10^6^ CFU/ml, which was adjusted by a spectrophotometer (Automatic Elisa Plate Analyser, Robonik, India)^16^. Group (1) remained as nonchallenged control negative, and group (2) was marked as control positive.

The health status of the challenged chicks was checked twice daily, and any clinical signs, mortalities and post-mortem (PM) lesions were recorded until the end of the experiment on the 12th day of chick age (10th day post inoculation). At the end of the experimental period, growth performance parameters; feed intake (FI), feed conversion ratio (FCR), body weight (BW) and body weight gain (BWG), were observed. At the end of the experiment, all of the remaining chicks were killed by cervical dislocation, and the ceca were collected aseptically from each chick and processed for *S.* Enteritidis isolation, which was confirmed by using slide agglutination tests with serotype-specific antisera^24^.

### Real-time PCR (RT‒PCR) for quantitative detection of *Salmonella* in cecal contents of chicks under experiment

The colonization of *S*. Enteritidis (CFU per gram of ceca) was evaluated using quantitative RT‒PCR; one gram of cecal contents was homogenized, and DNA was extracted from the prepared cecal samples using a QIAamp DNA Mini kit (Qiagen, Germany, GmbH). Briefly, 200 µl of the processed cecal content suspension was incubated at 56 °C for 10 min with 10 µl of proteinase K and 200 µl of lysis buffer. Then, 200 µl of ethanol (100%) was added to the lysate, and the samples were washed and centrifuged according to the manufacturer’s recommendations. DNA was eluted using 100 µl of elution buffer. The oligonucleotide primers and probes used for the *Salmonella* invA gene were supplied by Metabion (Germany) (Supplementary Table 1)^29^. DNA amplifications were performed in a final volume of 25 µl containing 3 µl of DNA template, 12.5 µl of 2 × QuantiTect Probe RT‒PCR Master Mix, 8.875 µl of PCR grade water, 0.25 µl of each primer (50 pmol conc.) and 0.125 µl of each probe (30 pmol conc.). Primary denaturation was performed for 15 min at 94 °C, followed by 40 cycles of denaturation for 15 s at 94 °C, annealing for 30 s at 49 °C and extension for 10 s at 72 °C using a Strata gene MX3005P real-time PCR machine, which had the ability to show the standard quantity with 3 decimals. *Salmonella* concentration within the examined samples was detected with CFU/gm. A known CFU/gm standard was serially diluted (tenfold), and then DNA was extracted from the last 5 dilutions separately and tested by RT‒PCR as mentioned above for the examined cecal content samples.

### Statistical analysis

Statistical analysis was performed using SPSS version 20. The One-Way ANOVA test was used to calculate the P value and determine the significant differences between experimental groups. A P value of 0.05 was considered statistically significant.

### Ethics approval and consent to participate

This protocol was performed by following the animal ethics guidelines and approved by Medical Research Ethics Committee of Mansoura University with code number [R/145]. Also, all methods are reported in accordance with ARRIVE guidelines. Consent to participate not applicable.

## Results

### Point prevalence and serotypes of *Salmonella*

Out of 200 samples, *Salmonella* was detected in 26 (13%) samples (Table 1), including 6 different serotypes (*S.* Enteritidis, *S*. Typhimurium, *S*. Santiago, *S*. Colindale, *S*. Takoradi and *S*. Daula). The predominant isolates were *S.* Enteritidis (l0) and *S*. Typhimurium (8), followed by *S*. Santiago (3), *S*. Colindale (2), *S*. Takoradi (2) and *S*. Daula (1).

**Table 1:** Antimicrobial resistance of isolated *Salmonella* serovars.

Serovars (no.)	DO	OT	S	N	SXT	FFC	CT	AMX	SAM
*S.* Enteritidis (10)	4	6	4	5	4	6	4	7	8
*S*. Typhimurium (8)	3	7	5	2	5	0	5	5	5
*S*. Santiago (3)	2	2	3	0	1	2	3	2	1
*S*. Colindale (2)	1	1	2	0	2	2	1	1	2
*S*. Takoradi (2)	1	1	1	1	0	1	1	2	2
*S*. Daula (1)	1	1	0	0	0	1	1	1	1
Total (26)	12	18	15	8	12	12	15	18	19
Resistant (%)	46.15%	69.23%	57.69%	30.77%	46.15%	46.15%	57.69%	69.23%	73.08%
Susceptible (%)	53.85%	30.77%	42.31%	69.23%	53.85%	53.85%	42.31%	30.77%	26.92%

*AMX* amoxicillin, *CT* colistin sulphate, *DO* doxycycline, *FFC* florfenicol, *N* neomycin, *S* streptomycin, *SAM* ampicillin/sulbactam, *SXT* sulfamethoxazole- trimethoprim, *OT* oxytetracycline.

### Antimicrobial susceptibility

Disc diffusion tests revealed that high resistance percentages were recorded with SAM (73.08%%), followed by AMX, OT (69.23%, for each), S and CT (57.69%, for each). Meanwhile, low resistance was recorded with DO, SXT, FFC (46.15%, for each) and N (30.77%). Most of the isolated *Salmonella*e showed multidrug resistance (Table 1). In addition, multidrug resistance (MDR) to three or more antimicrobial classes was detected in 24 out of 26 (92%) isolates with a high multidrug antibiotic resistance index (MARI) of 0.33–0.88 (Table 2). *Salmonella* serovars in this study showed 24 different MDR patterns (Table 2).

**Table 2:** Antibiotic resistant pattern profiles of isolated *Salmonella* strains.

Antibiotic pattern profiles	Antibiotics	No. of resistant isolates	Salmonella Serotypes	No. of resistance antibiotics	MARI
1	AMX, SAM, OT, SXT, FFC, CT, DO	1	*S.* Entritidis	7	0.77
2	AMX, SAM, N, OT, SXT, FFC, CT, DO	1	*S*. Entritidis	8	0.88
3	AMX, SAM, S, OT, FFC, CT, DO	1	*S*. Entritidis	7	0.77
4	AMX, SAM, OT, FFC, CT, DO	1	*S.* Entritidis	6	0.66
5	AMX, SAM, N, OT, SXT, FFC	1	*S.* Entritidis	6	0.66
6	AMX, SAM, N, S, OT, SXT	1	*S.* Entritidis	6	0.66
7	AMX, SAM, N, S, FFC	1	*S*. Entritidis	5	0.55
8	SAM, N, S	1	*S.* Entritidis	3	0.33
9	AMX, SAM, N, S, OT, SXT, CT	1	*S*. Typhimurium	7	0.77
10	AMX, SAM, S, OT, DO	1	*S*. Typhimurium	5	0.55
11	AMX, SAM, N, S, OT, OX, SXT	1	*S*. Typhimurium	7	0.77
12	AMX, OT, SXT, CT, DO	1	*S*. Typhimurium	5	0.55
13	AMX, SAM, OT, CT	1	*S*. Typhimurium	4	0.44
14	SAM, SXT, CT, DO	1	*S*. Typhimurium	4	0.44
15	S, OT, SXT	1	*S*. Typhimurium	3	0.33
16	S, OT, CT	1	*S*. Typhimurium	3	0.33
17	AMX, SAM, OT, FFC, CT	1	*S*. Daula	5	0.55
18	S, OT, SXT, FFC, CT, DO	1	*S*. Santigo	6	0.66
19	AMX, SAM, S, OT, FFC, CT	1	*S*. Santigo	6	0.66
20	AMX, S, CT, DO	1	*S.* Santigo	4	0.44
21	AMX, SAM, FFC, CT, DO	1	*S.* Takoradi	5	0.55
22	AMX, SAM, N, S, OT	1	*S*. Takoradi	5	0.55
23	AMX, SAM, S, FFC, SXT, CT, DO	1	*S.* Colindale	7	0.77
24	SAM, S, OT, FFC, SXT	1	*S*. Colindale	5	0.55

*AMX* amoxicillin, *CT* colistin sulphate, *DO* doxycycline, *FFC* florfenicol, *N* neomycin, *S* streptomycin, *SAM* ampicillin/sulbactam, *SXT* sulfamethoxazole- trimethoprim, *OT* oxytetracycline.

### The effect of in ovo inoculation of probiotic and florfenicol on egg hatchability and embryonic mortalities

On the day of hatching, the egg hatchability was estimated (number of hatched chicks/number of incubated eggs in each group), and the results (Table 3) revealed that the hatchability in groups 1, 2, 3, 4 and 5 was 86.7%, 93.3%, 100%, 100% and 100%, respectively. Embryonic deaths were recorded only in the non-inoculated (control) groups (1 and 2), and the inoculated groups (3, 4 and 5) showed no embryonic mortalities. The examination of dead embryos showed that the eggshells were sticky.

**Table 3:** The effect of in ovo inoculation of probiotic and florfenicol on egg hatchability and embryonic mortalities.

Group No	Treatment of groups	Embryonic mortalities	Egg hatchability
Group 1	No treatment	2/15 (13.3%)	13/15 (86.7%)
Group 2		1/15 (6.7%)	14/15 (93.3%
Group 3	Probiotic	0/15 (0%)	15/15 (100)
Group 4	Florfenicol	0/15 (0%)	15/ 15 (100)
Group 5	Probiotic and florfenicol	0/15 (0%)	15/15 (100)

### The effect of in ovo inoculation of probiotic and florfenicol on the control of *S.* Enteritidis infection and growth performance parameters in chicks under experiment post hatching

Post hatching, the chicks were housed separately, and the groups kept their previous numbers. Ten chicks post hatching from each group (2, 3, 4 and 5) were challenged orally at the 2nd day of age with *S.* Enteritidis (selected from the isolates of this study). Group 1 remained as a nonchallenged negative control. The health status of the challenged chicks was observed daily until the end of the experiment on the 12th day of age (10th dpc). Group 1 (nonchallenged) showed no clinical signs. Chicks in the positive control (group, 2) showed signs of ruffled feathers, loss of appetite, poor growth, closed eyes, diarrhea and pasted vent, which appeared at the 3rd day post inoculation. Group 4 showed signs of weakness, loss of appetite and diarrhea, which appeared on the 5th day post inoculation and lasted until the end of the experiment. Group 3 showed weakness and slight diarrhea, which started on the 6th day post inoculation. Few chicks in group (5) showed only slight diarrhea on the 7th day post inoculation, which lasted 2 days after that and then began to disappear. A total of 30% morality was recorded only in the positive control group. The PM examinations of the positive control revealed congestion of the internal organs, enteritis, enlarged liver with hemorrhages, unabsorbed yolk sac and pasted vent. Chicks in groups (3), (4) and 3 chicks in group (5) showed enteritis. No PM lesions were recorded in group (1).

Growth performance parameters (FI, BW, BWG, and FCR) were recorded at the end of the experiment on the 12th day post inoculation (Table 4). The mean values of BW and BWG in group 5 were significantly higher (p < 0.0001) than those in the other experimental groups. Meanwhile, the FI and FCR mean values were significantly lower in group 5 (p < 0.0001 and p = 0.035, respectively) than in the other groups.

**Table 4:** Means of body weight, body weight gain, Feed conversion ratio and feed intake of the chicks under experiment.

Groups No	Parameters			
	Body weight (BW) (g)	Body weight gain (BWG) (g)	Feed intake (FI) (g)	Feed conversion ratio (FCR)
Group 1	373.9 ± 0.99387	333.6 ± 0.991071	382.3 ± 0.667499	1.146 ± 0.005049
Group 2	179 ± 2.542964	138.8571 ± 2.610419	204.7143 ± 3.228592	1.147 ± 0.25385
Group 3	378.8 ± 0.38873	338.4 ± 0.426875	377.5 ± 0.372678	1.116 ± 0.000771
Group 4	372.5 ± 0.792324	332.2 ± 0.81377	381.1 ± 0.924362	1.118 ± 0.033006
Group 5	383.4 ± 0.452155	343.1 ± 0.433333	253.9 ± 0.498888	0.739 ± 0.0566
*P*-value	0.000	0.000	0.000	0.035

*Mean values expressed as mean ± SEM (mean ± standard error).

### Quantitative detection of *Salmonella* in cecal contents of chicks under experiment by using RT‒PCR

Cecal samples of the chicks in all groups were subjected to *S.* Enteritidis detection and confirmation with serotype-specific antisera. The results showed its detection in all chicks in the positive control group (2) and in all chicks of group 4, while it was detected in 9 chicks from group 3 and 3 chicks only from group 5. Group 1 (negative control) was negative for *S.* Enteritidis isolation (Table 5).

**Table 5:** Colonization of *S*. Enteritidis (CFU/ gm) in the cecal samples of the experimental chicks.

Code of samples	Group no	Results	Cut-off cycle threshold (CT)	Concentration of *S. Enteritidis* (CFU/g)
1	1 (Negative Control)	−	No CT	−
2	2 (Positive control)	+	14.25	2.307 × 10^6^
3		+	14.41	2.065 × 10^6^
4		+	15.39	1.048 × 10^6^
5	3 (treated with probiotic only)	+	28.04	1.660 × 10^2^
6		+	27.50	2.412 × 10^2^
7		+	26.00	6.809 × 10^2^
8		+	28.31	1.378 × 10^2^
9		+	26.14	6.180 × 10^2^
10		−	No CT	−
11		+	26.35	5.345 × 10^2^
12		+	27.44	2.515 × 10^2^
13		+	26.22	5.848 × 10^2^
14		+	29.16	7.652 × 10^1^
15	4 (treated with florfenicol only)	+	22.34	8.562 × 10^3^
16		+	17.82	1.952 × 10^5^
17		+	21.76	1.279 × 10^4^
18		+	20.69	2.681 × 10^4^
19		+	18.92	9.121 × 10^4^
20		+	20.30	3.511 × 10^4^
21		+	20.89	2.335 × 10^4^
22		+	21.52	1.510 × 10^4^
23		+	21.12	1.991 × 10^4^
24		+	21.46	1.956 × 10^4^
25	5 (treated with probiotics and florfenicols)	−	No CT	−
26		−	No CT	−
27		+	31.72	1.302 × 10^1^
28		−	No CT	−
29		−	No CT	−
30		+	31.42	1.603 × 10^1^
31		+	30.81	2.444 × 10^1^
32		−	No CT	−
33		−	No CT	−
34		−	No CT	−

**Figure 1:**
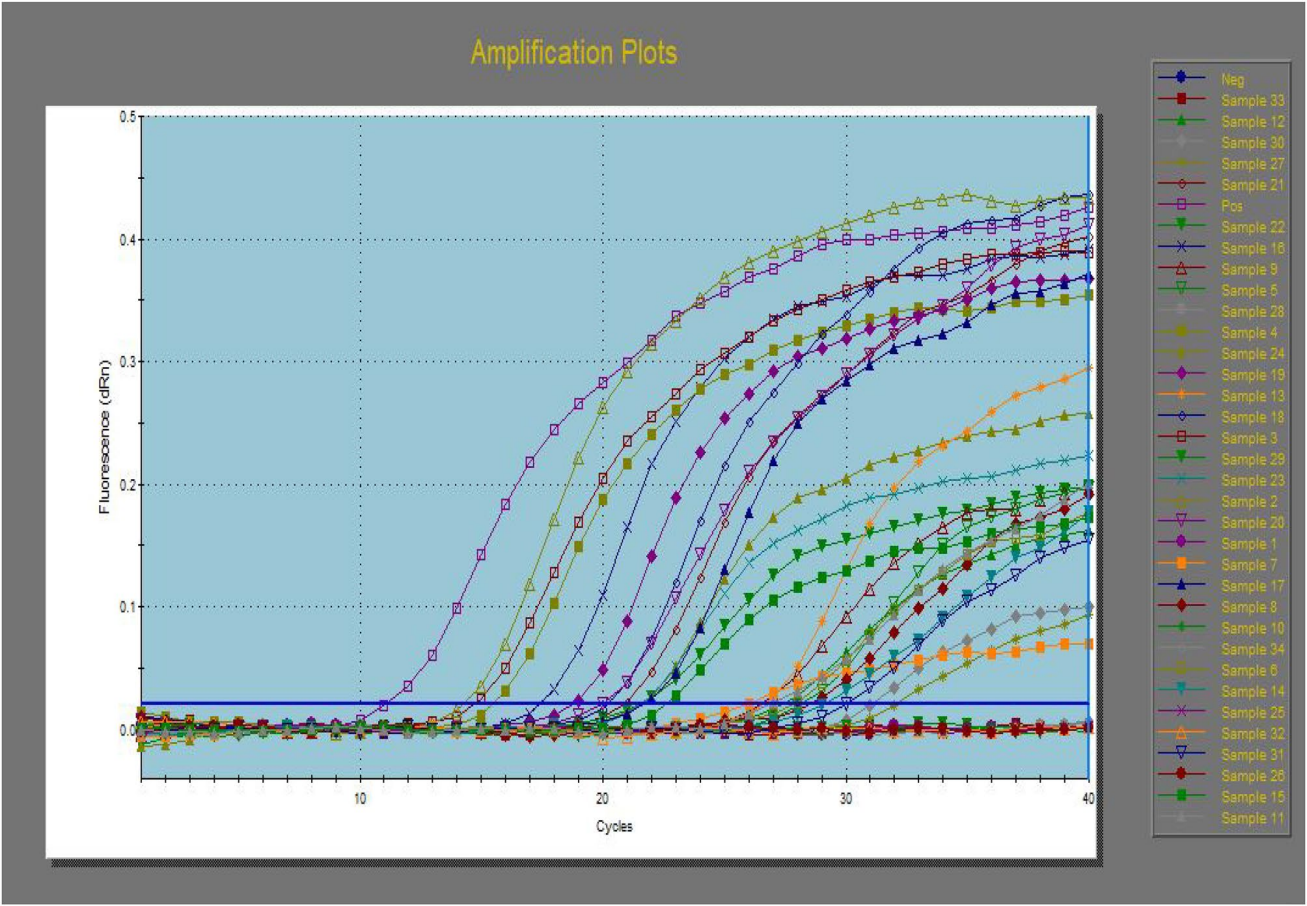
The amplification plots of multidrug resistant *S.* Enteritidis colonization in the examined cecal samples using RT- PCR.

The colonization of *S.* Enteritidis (CFU per gram of ceca) was evaluated using quantitative RT‒PCR. The results in Table 5 and Fig. 1 revealed that all chicks in group 4 showed colonization ranging from 8.562 × 10^3^ to 1.952 × 10^5^ CFU/gm. In group 3, the colonization ranged from 7.652 × 10^1^ to 6.809 × 10^2^ CFU/gm in 9 chicks, which was lower in comparison with groups (2) and (4). Group (5) showed lower colonization in only 3 chicks, and the other chicks showed no colonization. Although egg inoculation with the probiotic in group 3 showed slight improvement in the growth performance of the experimental chicks, it could not prevent clinical signs or *S.* Enteritidis colonization in the cecum. Meanwhile, the experimental chicks in group 5 were inoculated with probiotic and florfenicol showed good results; the growth performance parameters (FI, BW, BWG, and FCR) were improved when compared with other groups. The in ovo inoculation of both probiotic and florfenicol prevented posthatch *Salmonella* colonization in the cecum of 7 out of 10 chicks, and the remaining chicks showed a very lower colonization of *Salmonella*. Individual inoculation with probiotics and florfenicol did not prevent clinical signs or *Salmonella* colonization in the cecum.

## Discussion/Conclusion

In this study, *Salmonella* was isolated from diseased chickens in Dakahlia Governorate with a prevalence of 13%, and this percentage agreed to some extent with Elkenany et al.^30^, who isolated *Salmonella* from chickens at 13.5%. Previous studies included lower isolation rates than those recorded in this study^1,31^, Abd El-Ghany et al.^32^ and Ibrahim et al.^33^, who isolated *Salmonella* from chickens with percentages of 10.37%, 10.9%, 7.03% and 6.5%, respectively. However, a study conducted by Elkenany et al.^34^ isolated *Salmonella* at 32%, which represents a higher percentage than that recorded in this study. The variation in the *Salmonella* isolation rate between this study and other studies may be attributed to differences in geographical locations, sampling times and breeds. El-Sharkawy et al.^1^ found that higher infection rates may occur because of deficiencies in hygienic measures and biosecurity programs in chicken farms, which facilitate the spread of infections.

In our study, 6 serotypes of *Salmonella* were identified, including *S.* Enteritidis, S. Typhimurium, S. Santiago, S. Colindale, S. Takoradi and S. Daula. Another research study detected *S.* Enteritidis, *S*. Typhimurium, *S*. Infantis, *S.* Kentucky, *S*. Newport and *S.* Derby from broiler chickens^31^. Ramatla et al.^35^ isolated *S*. Typhimurium, *S*. Heidelberg, *S.* Enteritidis, *S*. Paratyphi B and *S*. Newport from chicken feces. *S.* Enteritidis causes paratyphoid cases in poultry and is associated with food-borne illness in human^6^. It was the most predominant serotype reported in this study, which agreed with^7^ Abd El-Ghany et al.^32^, Velasquez et al.^36^ and Elkenany et al.^34^. However, our results varied from Islam et al.^37^, who found that *S*. Pullorum was the most isolated serotype recovered from broiler chickens.

Concerning the disc diffusion tests performed in the present study, the results showed the presence of high resistance against SAM (73.08%), followed by AMX, OT (69.23%, for each), S and CT (57.69%, for each). Meanwhile, low resistance was recorded with DO, SXT, FFC (46.15%, for each) and N (30.77%). These findings were in near similarity with those of Hardiati et al.^38^, who reported that *Salmonella* isolated from chicken farms was resistant to OT and ampicillin (AM) with (75%), Islam et al.^39^, who showed resistance against AM (75%), N (50%) and DO (50%). Meanwhile, Velasquez et al.^36^ recorded a higher resistance of *Salmonella* isolates against S, and Alam et al.^40^ recorded resistance against AM and S of 82.85% and 77.14%, respectively. However, Wang et al.^41^ reported higher resistance against AM and CT with percentages of 97.6% and 51.2%, respectively. Most of the isolated *Salmonella* in this study showed multidrug resistance with high MDRI, which agreed with studies conducted by Elkenany et al.^34^, Xu et al.^42^ and Ibrahim et al.^33^.

After the establishment of *Salmonella*, primarily in the ceca^43^, it is very difficult to eliminate and reduce shedding without using specific antibiotics. Therefore, a crucial step in ensuring food safety is to reduce *Salmonella* colonization and environmental shedding^44^. Some studies have indicated that giving probiotic bacteria during the prenatal period of chickens can significantly reduce *Salmonella* colonization and fecal shedding^10,45^. Our results showed 100% egg hatchability, no gross abnormalities and an absence of embryonic deaths after in ovo inoculation of the probiotic with florfenicol. The hatchability was lowered in groups 1 and 2 (non-inoculated) with percentages of 86.7% and 93.3%, respectively. The recorded results agreed with those of Tavakkoli et al.^29^, who reported that the inoculation of florfenicol in eggs resulted in normal embryos with no gross abnormalities. Our results agreed to some extent with Rizk et al.^18^, who found that the inoculation of probiotic in ovo was associated with higher hatchability and lower embryonic mortalities. However, a research study conducted by Silva et al.^46^ reported that the hatchability of eggs inoculated with probiotics was 86.25%, and this finding was lower than that in our study.

Regarding the health status of the challenged chicks, clinical signs appeared on the chicks in the positive control group (2), group 3 and group 4. Few chicks in group 5 showed slight diarrhea, which disappeared rapidly after 2 days. Moralities were recorded only in the positive control group (30%) with PM lesions: internal organs congestion, enteritis, enlarged liver with hemorrhages, unabsorbed yolk sac and pasted vent. All chicks in groups 3, 4 and only 3 chicks in group 5 showed enteritis. In ovo inoculation of probiotic and florfenicol individually did not prevent the appearance of clinical signs and PM lesions. On the other hand, the majority of chicks in group 5, which were inoculated in ovo with both the probiotic and florfenicol, showed an absence of signs and PM lesions with significant improvements in growth performance parameters compared to the other groups. showed improvement. Rizk et al.^18^ found that eggs inoculated with probiotics showed significant improvement in growth performance.

Colonization of multidrug-resistant *S.* Enteritidis was counted in cecal samples of posthatched chicks using RT‒PCR, and the results showed that all chicks in groups 3 and 4 showed lower colonization than the positive control group. Group 5 showed lower colonization in only 3 chicks, and the other chicks showed no colonization. Our findings may be related to the initial probiotic colonization and presence of florfenicol, which stimulate the development and maturation of the immune system and prevent pathogenic bacteria from colonizing by competitive exclusion^11^. Our results agreed to some extent with De Oliveira et al.^16^, who reported a significant reduction in the number of *S.* Enteritidis*-*positive chicks when they were inoculated in ovo with *E. faecium* and continued to receive it in the diet. Meanwhile, Yamawaki et al.^47^ found that in ovo inoculation with *Lactobacillus acidophilus, L. salivarius or L. fermentum* did not prevent *S.* Enteritidis colonization in poultry ceca.

*Salmonella* was recorded from diseased broiler chickens with a prevalence of 13%. Six *Salmonella* serotypes were detected (*S.* Enteritidis, *S.* Typhimurium, *S*. Santiago, *S.* Colindale, *S.* Takoradi and *S*. Daula), and the most predominant serotype was *S.* Enteritidis (most of the isolates showed high MDRI). In ovo inoculation of probiotics with florfenicol not only improved egg hatchability and chick growth performance parameters but also prevented the infection of multidrug-resistant *S.* Enteritidis post hatching. Further studies should be applied to study the effect of both probiotics and florfenicol on *Salmonella* colonization in- ovo and after hatching.

## Supplementary Information

Supplementary Information.

## Data Availability

All data generated or analyzed during this study are included in this published article and its supplementary information files.
