# Supplementary material for: Synergistic influence of probiotic and florfenicol on embryonic viability, performance, and multidrug-resistant Salmonella Enteritidis in broiler chickens
Source: Sci Rep. 2023 Jun 14;13:9644. doi: 10.1038/s41598-023-36238-6 (PMC10267169; doi:10.1038/s41598-023-36238-6)
Supplement: Supplementary file 1 — Supplementary Information. [file 41598_2023_36238_MOESM1_ESM.docx]

**Supplementary table (1) Oligonucleotide primers and probes used in this study**

| **Gene** | **Primer/ probe sequence (5'-3')** | **Reference** |
| --- | --- | --- |
| *inv*A | GCGTTCTGAACCTTTGGTAATAA | **Daum *et al*., (2002).** |
|  | CGTTCGGGCAATTCGTTA |  |
|  | 5′-FAM-TGGCGGTGGGTTTTGTTGTCTTCT-TAMRA-3′ |  |
